# Motor Coordination Assessment in Autism Spectrum Disorder: A Systematic Review

**DOI:** 10.3390/diagnostics15172118

**Published:** 2025-08-22

**Authors:** Adriana Piccolo, Chiara Raciti, Marcella Di Cara, Simona Portaro, Rosalia Muratore, Carmela De Domenico, Alessia Fulgenzi, Carmela Settimo, Angelo Quartarone, Francesca Cucinotta, Angelo Alito

**Affiliations:** 1 IRCCS Centro Neurolesi “Bonino-Pulejo”, 98124 Messina, Italy; 2 Department of Human and Pediatric Pathology, University of Messina, 98124 Messina, Italy; 3 Physical Rehabilitation Medicine Department, University Hospital A.O.U. “G. Martino”, 98124 Messina, Italy; 4 Department of Biomedical, Dental Sciences and Morphological and Functional Images, University of Messina, 98125 Messina, Italy

**Keywords:** motor impairments, autism spectrum disorder, diagnosis, neuropsychiatric disorders

## Abstract

**Background/Objectives:** Motor difficulties are commonly reported in autistic individuals, but they are not currently part of the diagnostic criteria. A better understanding of how motor impairments are assessed in this population is critical to inform clinical practice and intervention. This systematic review aims to evaluate the existing literature on motor skill assessment in autistic children and adolescents, focusing specifically on studies that employed standardized and validated clinical motor assessment tools. **Methods**: Registered on PROSPERO (CRD42025637880), a systematic search was conducted on PubMed, Science Direct, and Web of Science until 31 December 2024. The review includes: (a) studies published in peer-reviewed journals; (b) randomized controlled trials (RCTs) and observational studies; (c) evaluations of motor difficulties using standardized and validated clinical assessments specifically designed to measure motor skills or coordination abilities; (d) participants diagnosed with ASD based on the Diagnostic and Statistical Manual of Mental Disorders (DSM-IV or DSM-5) or the International Classification of Diseases (ICD-9 or ICD-10); and (e) participants aged ≤18 years; **Results:** Twenty-two studies met the inclusion criteria. Most studies reported significant motor impairments across various domains, including balance, manual dexterity, and coordination. However, there was substantial variability in the severity of motor deficits and in the assessment tools used. Methodological heterogeneity limited direct comparison across studies. **Conclusions:** Motor impairments are common in autistic children and adolescents; however, current assessment tools show limitations and require adaptations. The findings underscore the need for autism-specific motor assessments to improve diagnostic accuracy and guide personalized interventions.

## 1. Introduction

Autism spectrum disorder (ASD) is a neurodevelopmental condition characterized by persistent deficits in communication and social interaction and limited, repetitive patterns of behavior, interests, or activities [1].

Symptoms must be present early in development and cause clinically significant impairments in social, occupational, and other important areas of functioning, where such impairments are not better explained by intellectual disability or global developmental delay [1]. Considering the heterogeneity and multifaceted nature of this neurodevelopmental condition, recent research has emphasized the importance of broadening the clinical perspective beyond core traits [2,3].

In recent years, increasing attention has been paid to additional features that, although not part of the core diagnostic criteria, may significantly affect functioning and development [4]. Among those, motor deficits can frequently be observed [5]. Indeed, pervasive gross motor abnormalities [6], balance difficulties, and postural instability [7] were frequently reported. However, globally, the majority of available evidence indicates that autistic individuals experience impairments across a range of motor domains, supporting the view that motor difficulties are a prevalent feature within the autistic population [8].

Data concerning early development indicates that approximately 50% of infants who later received an ASD diagnosis presented hypotonia, motor anomalies, and postural alterations within the first months of life [9,10]. Furthermore, around 40% of these individuals were later diagnosed with a motor coordination disorder, and in 20% of cases, a tendency to walk on tiptoe was also observed [9,10]. However, to date, findings in the literature remain somewhat inconsistent. Some studies report no significant differences in motor skills between autistic and non-autistic individuals, while others suggest that autistic individuals [11,12] may exhibit enhanced motor competencies [13].

A recent review highlighted that the motor and functional difficulties observed in autistic individuals are often consistent with those seen in developmental coordination disorder (DCD); however, only 3 out of 20 studies published since 2014 explicitly described these motor difficulties as DCD, suggesting that DCD remains substantially under-recognized in the autistic population [14].

Moreover, motor difficulties can also negatively impact other neuropsychological functions. Clinical research highlights that motor impairments can impact the ability to understand the actions of others [15]. Indeed, motor and social skills are intimately related. Literature data demonstrate that motor skills represent strong predictors of interpersonal coordination [16] and participation in social activities [17]. In ASD children, motor impairment can aggravate the clinical condition [18].

This interdependence suggests that motor difficulties may not only co-occur with challenges in social interaction but also contribute to their severity. Taken together, this body of evidence highlights the importance of investigating motor issues as potentially integral components of the autistic phenotype.

Despite much interest on the topic, there continues to be a clinical gap between the presence and diagnostic identification of motor deficits in ASDs [19]. To date, the assessment tools used to diagnose motor disorders in ASD children can be divided into macro-categories that include: home video analysis, parental reports, specific motor batteries, kinematic analysis of gait, and electronic balances [20]. However, there is currently no universally accepted ‘gold standard’ for assessing motor competence in this specific population. Autistic children often present with complex needs, including communication difficulties [21] and delays in response inhibition, cognitive flexibility/switching, and working memory [22]. This specific population may need to receive instruction and information differently than children with typical development and therefore may not be able to understand the assessment requirements in existing assessments.

The aim of this review is to understand what standardized measure of motor skills assessment can be most useful in identifying motor deficits in this population to improve early detection of motor deficits that may exacerbate social vulnerabilities [18,23]. Incorporating validated assessment instruments into routine clinical practice could enhance diagnostic precision, guide personalized interventions, and support the monitoring of rehabilitation outcomes, ultimately bridging a critical diagnostic gap and promoting better long-term trajectories for autistic individuals [19].

## 2. Materials and Methods

### 2.1. Information Source and Search Strategy

The protocol for this systematic review was registered in PROSPERO under the identifier: ID CRD42025637880. The systematic literature review was conducted following the PRISMA guidelines (Preferred Reporting Items for Systematic Reviews and Meta-Analyses) [24,25]. A completed PRISMA checklist is available in Supplementary Table S1. Searches were performed in the PubMed, Web of Science, and ScienceDirect databases until 31 December 2024. The search queries included keywords such as “motor” OR “coordination” OR “neuromotor” AND “assessment” AND “autism” for ScienceDirect and PubMed, while for Web of Science, the search strings were ALL = (motor) OR ALL = (coordination) OR ALL = (neuromotor) AND ALL = (assessment) AND ALL = (autism).

To extend the search, synonyms and MeSH terms corresponding to the main categories (autistic disorder and motor assessment) were utilized. In addition, the reference lists of included studies or reviews were searched to ensure that a comprehensive list of relevant articles was considered for inclusion.

### 2.2. Study Eligibility Criteria

To be included, studies had to meet the following criteria: (a) published in peer-reviewed journals; (b) randomized controlled trials (RCTs) with either parallel or crossover designs, uncontrolled clinical trials, and observational studies; (c) evaluation of motor difficulties using standardized and validated clinical assessments specifically designed to measure motor skills or coordination abilities; (d) participants diagnosed with ASD based on the Diagnostic and Statistical Manual of Mental Disorders (DSM-IV or DSM-5) [1] or the International Classification of Diseases (ICD-9 or ICD-10) [26,27]; (e) participants aged ≤18 years; and (f) articles published in any language.

Studies meeting these criteria were excluded: (a) not published in peer-reviewed journals; (b) meta-analyses, systematic reviews, case reports, case series, letters to the editor, comments, conference abstracts, and preclinical studies; (c) studies using non-standardized or non-validated assessments, or outcome measures not specifically designed to evaluate motor skills or coordination (e.g., kinematic analysis, voxel-based morphometry, dynamometers); (d) samples without a clearly defined ASD diagnosis; or (e) studies involving participants > 18 years of age.

### 2.3. Selection Procedures

Study selection was conducted by two blinded authors (A.P., C.R.); in case of disagreement, a third author (C.S.) was involved, who discussed the issue with the other authors and reached a consensus. If there was any doubt about inclusion, the article was moved on to the next stage. Initially, titles and abstracts were screened, excluding those not pertinent. After the screening stage, full-text articles were assessed for eligibility by two authors (A.P. and C.R.) independently, with 97.1% concordance. Discrepancies were resolved by the third author (C.S.).

### 2.4. Data Extraction and Evaluation

To analyze and categorize study characteristics, one author (F.C.) systematically coded all variables as categorical and clearly defined. The coding procedure incorporated both preliminary and final agreement checks to maintain consistency and reliability. After this initial phase, two independent authors (A.P. and C.S.) verified and aligned the classifications with predefined criteria, finally reaching full agreement on the coding and synthesis of the articles. Any inconsistencies identified during the interim reviews were addressed through discussion and resolved by consensus to ensure methodological consistency.

The data extraction process included: (a) general details of the study, including the first author, year of publication, and study design; (b) sample characteristics such as sample size, gender distribution, mean, standard deviation, and age range; (c) outcome measures; (d) motor test description; and (e) assessment of the reliability and feasibility of the motor assessments.

### 2.5. Feasibility and Reliability

As feasibility information was not consistently reported within the included studies, a standardized utility matrix was developed, based on criteria adapted from Beattie et al. [28] and Klingberg et al. [29], to independently appraise the practical aspects of each tool.

Feasibility was evaluated considering five key aspects: the time required to complete the full assessment, which indicates whether the tool can be administered within a short, moderate, or long time frame; the physical space needed to perform the assessment tasks, reflecting the practicality of use in typical school or clinical settings; the type and amount of equipment necessary, which influences costs and accessibility; the level of qualification required to administer the test, such as whether it can be conducted by a general teacher or demands a trained therapist or certified professional; and the extent of specific training needed for the administrator to conduct the assessment. Each of these aspects was rated on a standardized scale (1 = poor, 2 = fair, 3 = good, 4 = excellent, NR = not reported) as detailed in Table 1.

Reliability was assessed by examining inter-rater reliability, which reflects the degree of agreement between different raters observing or scoring the same performance; intra-rater reliability, which concerns the consistency of ratings given by the same rater on separate occasions; and test–retest reliability, which indicates the stability of results when the same test is administered to the same individuals at different times. Where available, these aspects were quantified using intraclass correlation coefficients (ICCs) or equivalent statistics, and the quality of the results was recorded as positive or not reported, as shown in Table 2.

### 2.6. Risk of Bias Assessment

The methodological quality of the included studies was evaluated using the Quality Assessment of Diagnostic Accuracy Studies (QUADAS-2) tool [52]. Two independent authors (A.A. and M.D.C.) conducted the assessment, and any discrepancies were resolved through discussion with a third author (F.C.). The QUADAS-2 tool examines the risk of bias and applicability issues in four areas: (1) patient selection: the recruitment strategy was considered, and the risk of selection bias was assessed; (2) index test: how the motor coordination measures were conducted and interpreted was evaluated, particularly with regard to blinding with respect to the reference standard; (3) reference standard: the validity and independence of the diagnostic procedures used to establish an ASD diagnosis were assessed; and (4) flow and timing: whether all participants were subjected to the same reference standard and included in the final analysis was addressed, and whether the time interval between the index test and the reference standard was acceptable. Each domain was rated as having a low, high, or unclear risk of bias and applicability concerns.

## 3. Results

**Figure 1:**
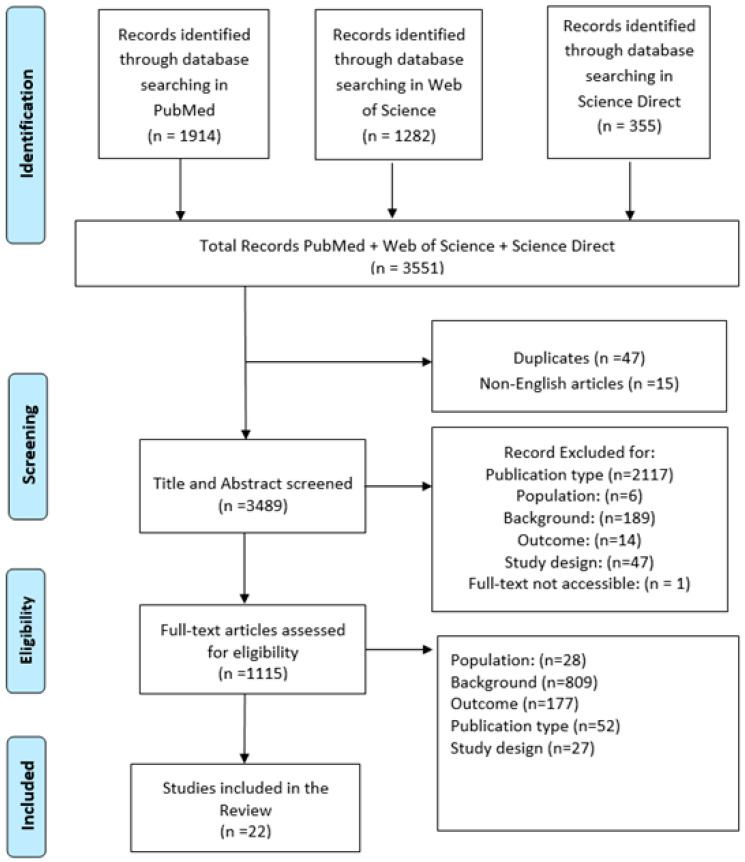
Preferred Reporting Items for Systematic Reviews and Meta-Analyses (PRISMA) flow diagram for study selection.

A total of 3551 records were identified through database searches (PubMed, Web of Science, and Science Direct). After the removal of 47 duplicates and 15 non-English articles, 3489 records were screened by title and abstract. Of these, 1115 full-text articles were assessed for eligibility. Following this phase, 22 studies were included in the final review (Figure 1). Articles were excluded based on the following criteria: 52 for publication type, 809 for background irrelevance, 27 for study design, 177 for inappropriate outcome, and 28 for unsuitable population. Extracted data included study design, participant characteristics (e.g., age and diagnostic group), and outcome measures. The selected studies were also evaluated for the reliability and feasibility of the motor assessment tools used.

The 22 included studies assessed motor skills using standardized tests (see Table 3 for more details).

Sample characteristics and range of age differ significantly among these studies. Sample sizes ranged from n.24 to 15,573 (mean 1254.6), with participants’ ages ranging from 0 to 24 months for the youngest sample to 18 years in the oldest. All studies included in the review were observational in design; none were RCTs.

The majority of studies (N = 11; 54.5%) included only participants with typical development (TD) compared with those with autism spectrum disorder (ASD) [31,32,35,37,38,39,41,44,46,49,51]. Several other studies involved mixed samples. Specifically, one study [40] examined individuals with ASD, TD, and born preterm; another study [42] included participants with ASD, TD, and mental retardation; two studies [34,45] included individuals with ASD, TD, and attention-deficit/hyperactivity disorder (ADHD); one study [43] focused on participants with ASD and global developmental delay (GDD); another [33] included individuals with ASD and developmental coordination disorder (DCD); one study included individual with ASD, DCD, and TD [48]; and one study [50] investigated individuals with ASD and motor developmental delay (MDD). Finally, three studies [30,36,47] included only participants diagnosed with ASD.

The sample characteristics of the study design, objectives, outcome measures, and main results are summarized in the Table 4.

The analysis of the 22 included studies shows that 20 authors (90.91%) conducted their studies using a single rating scale to assess motor skills in ASD [30,31,33,34,35,36,37,38,39,40,41,43,44,45,46,47,48,49,50,51], while the other two studies (9.09%) used several scales simultaneously [32,42].

In total, five standardized tests were described and used in the included articles to assess motor coordination: Movement Assessment Battery for Children Second Edition (MABC-2) [53], Bruininks–Oseretsky Test of Motor Proficiency, Second Edition (BOT-2) [54], Peabody Developmental Motor Scales—Second Edition (PDMS-2) [55], Test of Gross Motor Development (TGMD) [56], and Alberta Infant Motor Scale (AIMS) [57]. They will now be analyzed individually in order to better understand their limitations and strengths.

### 3.1. Movement Assessment Battery for Children

From the total of studies analyzed in this review, the most used and widespread battery appears to be the Movement Assessment Battery for Children. A total of 12/22 (54.5%) studies included the MABC-2 tool. However, only three studies used this instrument as the sole outcome measure [34,39,48]. Overall, they examined participants whose ages ranged from 7.96 to 12.48 years, with a mean age of 10.41 years (SD = 1.26). The MABC-2 tool was applied in various experimental contexts. Several studies [32,34,35,38,39,45,48,51] (36.4%) compared autistic children with typically developing peers. In other cases, the comparison involved autistic children, typically developing children, and children with DCD [48], or autistic children and children with DCD [33]. Studies by Ament et al. [34] and De Francesco et al. [45] also included a third group consisting of ADHD children, alongside the ASD and TD groups. All studies that administered the complete version of the MABC-2 assessed three standard motor domains: manual dexterity, aiming and catching, and balance.

Consistently, findings showed that autistic children and adolescents exhibit significantly greater motor impairment, particularly in balance, aiming, and catching tasks, compared with TD peers. Studies by Ament et al. [34], Liu et al. [39], and De Francesco et al. [45] confirmed this difference across different diagnostic groups (ASD, ADHD, TD), while research by Bricout et al. [38] and Odeh et al. [32] used MABC-2 in combination with other tools to define specific motor profiles in ASD, revealing marked impairments in complex and balance-related tasks. Notably, Odeh et al. also found significant discrepancies between MABC-2 and BOT-2 scores for the same subtests, highlighting the importance of appropriate test selection. Specifically, significant differences were found between the scores obtained with MABC-2 and BOT-2 in equivalent subtests, particularly in the aiming and grasping tasks. In these domains, MABC-2 proved to be less sensitive in detecting group differences. Faber et al. [35] demonstrated a robust association between MABC-2 motor outcomes and deficits in visuomotor integration, with 75% of ASD participants scoring in the impaired range. In a large sample study of Green et al. [30], 79% of ASD children showed movement impairments per MABC, with greater severity linked to intellectual disability. Miller et al. [33] found that over 97% of ASD cases scored below the 16th percentile, supporting high DCD co-occurrence. Finally, Martel et al. [48] used MABC-2 to differentiate ASD from DCD profiles, identifying different motor control patterns that underscore the need for individualized rehabilitation strategies.

Five studies [30,33,41,44,45] used MABC and the Developmental Coordination Disorder Questionnaire (DCDQ) as an outcome measure, reflecting a growing interest in triangulating objective clinical assessments with parent-reported screening tools. Although DCDQ offers practical advantages such as ease of administration and cost-effectiveness, its diagnostic accuracy showed moderate agreement with MABC. For example, Green et al. [30] demonstrated that while MABC identified 79% of children with ASD with a marked motor disorder, the DCDQ tool reported a sensitivity of 66% and a specificity of 75% when compared with the same sample. Similarly, in the study of Miller et al. [33], both the MABC and DCDQ tools were used to assess motor function; however, risk ratings obtained via the DCDQ did not significantly align with those of the MABC-2, either in the ASD or DCD group, suggesting that parent-reported concerns may not always reflect standardized motor performance outcomes. In the study by Crippa et al. [41], although both the MABC-2 and DCDQ clearly differentiated children with ASD from typically developing peers, no direct association was found between the two measures. While the authors of study [44] did not report direct comparisons between the two measures, in the study by De Francesco et al. [45], the DCDQ and MABC-2 tools captured different motor dimensions, with caregiver reports predicting autism compared with children with autism spectrum disorder, and the MABC-2 subscales in particular targeted and captured the distinction between autism and ADHD, supporting their complementary value.

The feasibility analysis shows that the MABC-2 is positively rated in terms of the time required to complete the test, which is considered excellent. This means that the test can be administered in a relatively short time compared with other motor assessment tools. The space required is also rated excellent, indicating that the test can be conducted in environments with limited space. Regarding equipment, MABC-2 receives a fair rating. This suggests that while some specific equipment is needed, it is not particularly complex or expensive, but it must still be available for proper test administration. The use of the MABC-2 is limited only by the qualification required for the administrator, meaning that only professionals with specific training can properly administer the test. However, the training required to use the MABC-2 is considered fair. It requires some preparation for the administrator, but it is not deemed overly complex or demanding. Only one study, Odeh et al. [32], assessed the reliability of the MABC-2 battery, showing high intra-rater reliability with an ICC ranging from 0.988 to 0.994 (see Table 2). This indicates that the results obtained using the MABC-2 were consistent and reliable when administered by the same evaluator at different times, minimizing variability related to administration.

### 3.2. Bruininks–Oseretsky Test of Motor Proficiency Second Edition and BOT-SF

A total of N 5/22 (22.7%) studies considered BOT-2 in the outcome measures, examining an age range between 5 and 12 years, with a mean age of 8.80 years (SD = 2.68).

In studies using the BOT-2 [31,32,37,49,51], the instrument was administered to samples consisting of both autistic children and typically developing peers, allowing for direct group comparisons.

The specific domains assessed varied depending on whether the full or abbreviated version of the test was used. Studies by Odeh et al. [32] and Kalfirt et al. [49] administered the full battery, covering all four major domains: fine manual control, manual coordination, body coordination, and strength/agility. In contrast, studies by Kaur et al. [31], Alsaedi et al. [37], and Martin-Diaz et al. [51] used selected subtests or the abbreviated version, typically assessing fine motor skills, balance, coordination, and strength, although not all components of gross motor skills were included.

Kaur et al. [31] found that both high-IQ and low-IQ ASD groups showed significantly worse performance than TD peers in gross and fine motor skills, with the low-IQ group being particularly impaired in body coordination and overall BOT-SF scores. Similarly, Odeh et al. [32] reported lower scores in ASD participants across BOT-2 subtests, particularly in upper limb coordination, strength, and agility. Alsaedi et al. [37] confirmed these findings in a large ASD sample, showing broad impairments in all BOT-SF subtests, with strength emerging as the most affected domain (Cohen’s d = 2.66), although some age-related improvement in motor skills was noted. Kalfířt et al. [49], using the full BOT-2, highlighted significant deficits in hand coordination, running speed, agility, and strength in ASD individuals, whereas balance and bilateral coordination were relatively preserved. Lastly, Martín-Díaz et al. [51] observed large effect sizes in BOT-2 SF total and subtest scores, with 84% of ASD participants performing below average, particularly in strength, manual dexterity, and fine motor precision. These findings collectively support BOT-2 and BOT-2 SF as sensitive tools for detecting specific motor coordination impairments in ASD populations.

Three authors of five studies [31,37,49] used the BOT-2 as the sole motor assessment tool in their study.

Two studies [32,51] compared the BOT-2 with other motor assessment tools. The study by Odeh et al. [32] reported divergent results between the BOT-2 and the MABC-2 on equivalent subtests, particularly for aiming and grasping, suggesting possible differences in sensitivity (see Section 3.1). The study by Martin-Diaz et al. [51] found weak to moderate correlations between BOT-2 scores and other measures such as the TUG test and the Pediatric Balance Scale, indicating that these tools may assess distinct aspects of motor performance.

None of the studies using the BOT-2 reported substantial limitations of the tool itself. One study [32] also highlighted feasibility-related challenges. Although the BOT-2 instrument provides comprehensive motor coverage and includes visual support that may benefit autistic children, its full administration was described as time-consuming and potentially fatiguing. The number of items, together with attentional and behavioral demands, were noted as possible obstacles in clinical settings.

Reliability of the BOT-2 tool was explicitly examined in studies of Kaur et al. [31] and Odeh et al. [32]. Kaur et al. [31] assessed inter-rater reliability and reported an ICC of 0.99 for both gross and fine motor scores, indicating excellent consistency between different raters. Similarly, Odeh et al. [32] evaluated inter-rater reliability using videotaped assessments and found ICC values ranging from 0.970 to 0.996 across BOT-2 subtests, confirming high measurement stability.

Regarding feasibility, BOT-2 received fair scores for both administration time and space requirements and a good rating for equipment needs. Although qualifications were not reported, the training required was rated as fair. These scores suggest that while the BOT-2 tool is manageable in most clinical settings, its administration may still present practical challenges, particularly in terms of duration and space availability.

### 3.3. Peabody Developmental Motor Scales

Four studies (18%) investigated motor performance in young ASD children using a standardized PDMS battery. All four studies [36,40,42,43] were observational; among them, studies [36,40] had an exploratory aim. The studies’ samples take into consideration an age range between 1 and 10 years; within this range, the most represented average age is 5.5 years. Of the studies using the PDMS-2, only one study [36] focused exclusively on autistic children. In contrast, Yang et al. [40] included autistic children, TD peers, and premature infants with low birth weight. Study [42] compared ID, TD, and autistic children, while only study [43] compared autistic children and children with global developmental delay (GDD).

The PDMS assesses both gross and fine motor skills through six subtests, including reflexes, stationary control, locomotion, object manipulation, grasping, and visual–motor integration.

Studies used all subtests within the test, except the study by Provost et al. [43], which did not consider the stationary and grasping subtests.

Globally, these studies confirm significant motor impairments in ASD. Specifically, Fulceri et al. [36] applied artificial neural networks (ANNs) and found significant correlations between PDMS-2 scores and both restricted and repetitive behaviors (ADOS-RRB) and language abilities (ADOS Module 1), suggesting that motor deficits may be developmentally linked to core ASD symptoms. Similarly, Yung et al. [40] reported that ASD children born at term scored significantly lower across all PDMS-2 motor quotients compared with both TD and those born preterm with very low birth weight, with most ASD participants falling into the “Below Average” or “Poor” classification. Likewise, Yang et al. [40] confirmed that ASD children had lower motor performance and more behavioral issues than both comparison groups. Interestingly, Vanvuchelen et al. [42] further demonstrated a significant correlation between general motor skills and gestural imitation abilities, suggesting that motor difficulties may contribute to imitation deficits often observed in ASD. Collectively, these findings show the PDMS-2 as a valid tool for identifying and characterizing motor difficulties in young children with ASD, particularly in relation to other developmental domains. Conversely, Provost et al. [43] found no significant differences between ASD and the GDD-specific diagnostic group but emphasized the utility of PDMS-2 in evaluating motor development and stressed the need to adapt its administration for children with comprehension difficulties.

Of the studies reviewed, only one included a direct comparison between the PDMS-2 and another motor assessment tool. In the study by Vanvuchelen et al. [42], both the PDMS-2 and the MABC tools were administered. The results were consistent between the two tools, indicating general motor deficits in autistic participants and supporting the validity of the findings.

One study [40] noted that the PDMS-2 tool may have limitations in capturing more subtle or segmental motor behaviors, which are important for a comprehensive assessment of motor skills in young autistic children.

No study has evaluated the feasibility of this tool; our utility matrix analysis considers the administration time of the PDSM to be quite long, which could make it less practical to be completed. The space requirement is moderately practical. It can be administered by various professionals with moderate training requirements (see Table 1). Reliability was assessed in only one study [43] of the four studies that used the PDSM and indicated the inter-rater reliability of the PDMS-2 with an extremely high level of reliability between raters.

### 3.4. Test of Gross Motor Development

A total of 2 out of 22 studies (9%) employed the TGMD-2 or TGMD-3 to assess fundamental motor skills in autistic children. The studies that included the TGMD test [46,47] involved participants aged between 4 and 18 years, with a mean age of 11 years. Both studies administered all domains of the TGMD, including the locomotor and ball/object control subtests, to provide a comprehensive assessment of gross motor skills, and they included two groups of participants: autistic children and TD.

Study [46] used the TGMD-3 test to assess fundamental motor skills in autistic children aged 4–10 years compared with TD peers. Results showed that autistic children scored significantly lower on locomotor skills and object control skills compared with TD children. Comparing autistic children with three groups based on specific developmental variables (chronological age, motor skills, and cognitive development), study [47] using the TGMD-2 test showed that when matched by chronological age, autistic children performed significantly worse than peers. However, when matched by motor skill level, they performed similarly to TD children approximately half their age. In contrast, when matched by cognitive ability, the autistic group still showed more impaired motor performance than expected. These findings suggest that the motor deficit in autistic children is structural and cannot be explained by cognitive delay or age alone.

While the study by Allen et al. [46] did not report any direct limitations regarding the TGMD-3 test, the study by Staples and Reid [47] noted that the TGMD-2 test may not capture the qualitative aspects of movement execution, such as awkwardness or poor coordination, which are often observed in autistic children. This limitation suggests that the TGMD test may underestimate clinically relevant motor difficulties in this population. Additionally, the study by Allen et al. [46] highlights the need for personalized support during assessment to best meet the needs of this population.

Neither of the two studies compared the TGMD test with other motor assessment tools.

The reliability of the instrument was assessed in the study by Allen et al. [46], showing exceptionally high reliability for both versions: TGMD-3 Traditional (ICC = 0.95) and TGMD-3 Visual (ICC = 0.95) (see Table 2).

The feasibility of both the TGMD-2 and TGMD-3 tests was rated as moderate. Both versions received good scores for time and equipment, while showing more limited ratings in other categories such as space and training.

### 3.5. Alberta Infant Motor Scale

The only study [50] using the AIMS test (4.5%) was conducted on 240.299 children registered at one health care organization in Israel and born between 2011 and 2017. The analysis focused on the subgroup of children who received at least one developmental physiotherapy session before age two due to suspected motor delays in order to retrospectively evaluate whether AIMS could help identify infants at risk of ASD before the emergence of typical social and communication symptoms. The sample was composed of an ASD population and a motor developmental delay (MDD) group. The study used all 58 items of the AIMS, covering the four standard motor domains: prone, supine, sitting, and standing. The results identified motor delays in over 87% of autistic children and MDD, suggesting that AIMS could be a useful screening tool to identify early children at risk of ASD based on their motor development. The study reported no specific limitations or concerns regarding the use of AIMS, and the tool was applied without criticism. Despite the large sample enrolled, this is the only study using this tool, thus reducing the generalizability of the results.

The study does not directly report reliability and feasibility coefficients. However, this tool can be considered highly feasible for clinical use due to the brevity of administration, ease of application in different environments, and low need for specialized equipment (see Table 1).

### 3.6. Feasibility and Reliability

Based on the feasibility scores summarized in Table 1, the standardized tools generally demonstrated good feasibility across most criteria, although there were some notable differences. Overall, the AIMS test achieved the highest feasibility scores and rated excellent (score 4) across nearly all categories, including time required, space, and equipment availability. Specifically, equipment and time criteria were the least critical factors for most tools: for example, five of six tools (83%) scored 3 (good) or 4 (excellent) in the equipment domain, indicating they required minimal or easily accessible materials. Similarly, time required for administration was generally rated good or excellent in 66% of studies (four of six tools). Conversely, qualification and training requirements emerged as the most critical feasibility aspects. Notably, three of six instruments (50%) received only fair or poor scores (1–2) for the qualification criterion, suggesting that these tests may require administration by trained professionals rather than general practitioners. Training requirements also showed limitations: all the instruments (100%) were rated fair, highlighting the need for specific training to ensure proper administration and scoring.

In terms of reliability (Table 2), only 5 out of 22 studies (22.7%) reported complete inter-rater or intra-rater reliability statistics. Among these, results show high overall consistency of measurement for the standardized instruments used. The BOT-2 and MABC-2 tests demonstrated excellent inter-rater and intra-rater reliability, with ICCs consistently above 0.97, indicating high agreement. The PDMS-2 and TGMD-3 studies also showed that good reliability indicators were reported, with ICCs above 0.95.

### 3.7. Quality Assessment

A methodological quality evaluation was conducted to assess the consistency and accuracy of the evidence presented by the included studies. This assessment focused on risk of bias and applicability concerns, providing a detailed overview of the strengths and limitations of the study designs in key areas.

Patient selection was the most frequent source of bias, with 86% of studies rated as high risk. The index test showed greater variability, but only 9% of studies had high risk, and 50% were considered low risk. For the reference standard, 55% of studies were rated as low risk, and just 14% as high. In the flow and timing domain, 45% of studies were rated as low risk, while 23% showed high risk, and the rest were unclear or inconsistently reported. Applicability concerns were most notable in the patient selection domain, where only one study was rated as low concern, while 36% were high and 59% were unclear. In contrast, the index test domain demonstrated strong alignment with the review objective, with 82% of studies showing low concern. For the reference standard, half of the studies had low applicability concerns, whereas 27% were high and 23% were unclear.

**Figure 2:**
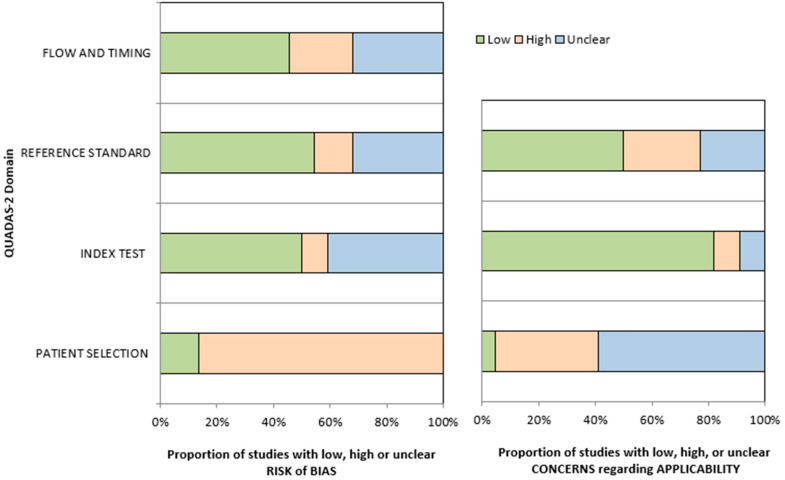
Risk of bias and applicability concerns by QUADAS-2 domain. The proportion of studies with a low, high, or unclear risk of bias (**left**) and applicability concerns (**right**) is shown across the four QUADAS-2 domains.

A summary of these findings is presented in Figure 2 and Table 5.

## 4. Discussion

Motor impairments are highly prevalent in ASD individuals, often affecting gait [58], manual dexterity [59], static and dynamic balance, and goal-directed motor skills [60]. Despite their frequency and clinical relevance, motor deficits are not considered diagnostic criteria for ASD; for this reason, they are often under-diagnosed, and they are frequently attributed to a comorbid diagnosis of DCD, resulting in dual diagnoses [61]. However, evidence increasingly suggests that motor symptoms may be extremely frequent in ASD with significant implications for cognitive, social, and adaptive functioning [39,62]. Despite this increasing evidence, there is a lack of specific assessment tools for this population.

Motor difficulties in ASD are often evident early in development and may persist in adolescence, contributing to reduced participation in social and physical activities and increasing the likelihood of behavioral challenges [9,63]. Only one study [37] included in this review reported a modest improvement in motor performance with age, supporting that early identification and intervention are critical. In this context, AIMS [5] emerges as a valuable early screening tool. In a retrospective study involving over 240,000 children, more than 87% of those later diagnosed with ASD showed early motor delays. Specifically, the AIMS tools may be particularly advantageous in clinical practice due to its brief administration time, minimal equipment requirements, and adaptability to various settings.

Across the reviewed literature, most studies assessed motor functioning in ASD using standardized tests such as the MABC-2, BOT-2, PDMS-2, and TGMD-2/3 [30,32,34,35,37,39,41,45,48,49,51]. A few works aimed specifically to evaluate which instruments are most appropriate for this population [39,46], while others used a combination of tools to mitigate the limitations of individual tests. A key finding of this review is the heterogeneity in the instruments used and the motor domains assessed, which highlight the complexity of motor coordination assessment in autistic individuals.

Among the most widely used instruments, the MABC-2 tool has emerged as one of the most frequently used and psychometrically robust tools. The test assesses manual dexterity, balance, and aiming/catching, allowing a comprehensive motor profile. Green et al. [30] reported that 79% of autistic children showed definite motor impairment, particularly in timed tasks, suggesting that motor difficulties in ASD may be linked to deficits in temporal processing. Ament et al. [34] further confirmed that visual–motor tasks requiring rapid adjustments, such as aiming and catching, were particularly challenging. Similarly, Liu et al. [39] found that 77% of children with ASD scored in the “red zone” of MABC-2, indicating severe impairment. Faber et al. [35] and Crippa et al. [41] corroborated these results, highlighting persistent deficits across all subscales. Martel et al. [48] and De Francesco et al. [45] used the MABC-2 tool to differentiate ASD from DCD or ADHD, demonstrating high diagnostic accuracy, especially in subtests assessing manual dexterity and visual–motor integration.

The BOT-2 instrument was also valued for its ability to assess both fine and gross motor skills. Kaur et al. [31] and Odeh et al. [32] demonstrated its ability to distinguish motor profiles in ASD subgroups, including those with high versus low cognitive functioning. BOT-2 effectively captured deficits in strength, manual coordination, and agility. Martín-Díaz et al. [51] reported that 84% of autistic children fell into the “below average” or “well below average” range, with large effect sizes. Despite its strengths, the length of the BOT-2 tool, the complexity of some items, and the need for specialized equipment may limit its feasibility, particularly in children with low frustration tolerance. However, the use of visual aids, such as photographs included in the administration protocol, may improve comprehension and compliance in autistic children [64].

PDMS-2 was particularly useful for the assessment of younger children, offering a detailed assessment of both fine and gross motor domains. Fulceri et al. [36] identified consistent motor deficits in ASD preschoolers that correlated with restricted and repetitive behaviors and lower expressive language skills. Yang et al. [40] found that autistic toddlers showed poorer PDMS-2 scores and greater behavioral difficulties than preterm or TD peers. Vanvuchelen et al. [42] emphasized the relationship between general motor deficits and imitation difficulties, suggesting a perceptual–motor basis for impairments in social communication. Provost et al. [43] highlighted the value of PDMS-2 in establishing developmental motor profiles, particularly when adapted for children with attentional or behavioral challenges. A common limitation noted was the duration of administration and the need for flexible procedures in ASD populations.

The TGMD-2 and TGMD-3 tools, which focus on gross motor skills, were found to be reliable and valid tools for assessing locomotor and object control skills in ASD. Allen et al. [46] demonstrated high internal consistency and test–retest reliability, with improved performance when visual support was included. Staples and Reid [47] confirmed persistent gross motor delays in autistic children compared with matched typically developing peers, even into later childhood.

Although the DCDQ is a questionnaire-based tool and was not a primary focus of this review, it was used alongside diagnostic instruments in many of the studies examined [30,33,41,44,45]. Data from these studies indicates that, in such contexts, DCDQ can effectively classify motor functioning profiles in children with ASD, ADHD, and TD. It showed particularly high accuracy in distinguishing ASD from TD. However, despite its efficiency and ease of administration, its results may be influenced by caregiver bias and subjective interpretation [33,45].

Overall, the findings of this systematic review emphasize that while many instruments demonstrate validity and utility in assessing motor impairments in ASD, each has specific limitations. The choice of tool should be guided by the child’s age, cognitive profile, and behavioral characteristics and by the goals of the assessment. Tests such as MABC-2 and BOT-2 offer comprehensive evaluations but may be time-consuming or require adaptations. Instruments like AIMS or DCDQ may be more feasible for screening but lack the depth of standardized performance assessments.

The overall methodological quality of the studies included in this review reveals significant limitations in the existing literature on motor assessment in individuals with ASD. Most studies exhibited a high risk of bias, particularly with regard to participant selection, as they often relied on convenience sampling and lacked detailed diagnostic confirmation of ASD. These methodological weaknesses raise concerns about the generalizability of the findings and the validity of comparisons between groups. Nevertheless, the majority of studies used standardized, clinically relevant motor assessment tools that were generally applied consistently and appropriately, thus supporting the relevance of the selected index tests to the aims of this review. Applicability issues were particularly apparent in the definition and recruitment of populations, whereas the execution of the tests themselves aligned well with the intended clinical context. These findings suggest the need for future diagnostic accuracy studies that adopt more rigorous recruitment procedures and validated reference standards in order to better support the use of motor measures in ASD profiling.

Furthermore, given the high prevalence and early onset of motor difficulties in ASD [65], there is a clear need for the development of an ASD-specific motor assessment tool. As highlighted by Braconnier & Siper [66] and Fay et al. [67], adaptations during test administrations such as providing additional demonstrations or modifying task demands are more common in ASD than in other neurodevelopmental disorders, potentially undermining the standardization and validity of existing instruments. A tailored tool should integrate multimodal assessment domains, including fine and gross motor skills, sensory integration, visuomotor coordination, and social-pragmatic motor abilities. This contribution could not only improve diagnostic accuracy but also facilitate the identification of individualized rehabilitation strategies, overcoming the limitations of currently used instruments. The development of such a test would represent a significant step toward greater inclusiveness and targeted support for this population.

### Limitations

One limitation of this review is the small number of studies specifically aimed at evaluating the feasibility of a motor assessment measure in ASD individuals. Since most of the studies included had different primary objectives, this may have affected the ability to systematically compare and identify the most appropriate assessment tools for this population. However, despite the variability in study aims and methodologies, the findings offer valuable insights into the range of instruments used and emphasize the need for further research focused on feasibility and reliability and standardization in this population.

## Figures and Tables

**Table 1:** Feasibility scores for motor assessments.

Assessment Measure	Time	Space	Equipment	Qualification	Training
MABC-2	3	3	2	1	2
BOT-2	2	2	3	NR	2
PDMS-2	1	3	3	1	2
TGMD-2	3	2	3	NR	2
TGMD-3	3	1	3	NR	2
AIMS	4	4	4	2	2

Legend: 1, poor; 2, fair; 3, good; 4 excellent; NR, not reported.

**Table 2:** Reliability measurement for the studies.

References		Reliability Measurement	Result	Quality of Results
[30]	Green, D. et al. (2009)	Not reported	Not reported	Not reported
[31]	Kaur, M. et al. (2018)	Inter-rater reliability Intra-rater reliability	BOT: (inter) Gross motor ICC = 0.99 Fine motor ICC = 0.98 (intra) Gross motor ICC = 0.99 Fine motor ICC = 0.98	+ + + +
			SIPT-BMC: (inter) Rhythmicity ICC 0.96, Mirroring ICC 0.99, Overflow ICC0.80, Time ICC 0.80. (intra) Rhythmicity ICC = 0.97, Mirroring ICC = 0.97, Overflow ICC = 0.95, Time ICC = 0.99	
[32]	Odeh, C.E., et al. (2022)	Intra-rater reliability	MABC-2: ICC = 0.988–0.994 BOT-2: ICC = 0.970–0.996	+ +
[33]	Miller, HL. et al. (2021)	Not reported	Not reported	Not reported
[34]	Ament, K. et al. (2015)	Not reported	Not reported	Not reported
[35]	Faber, L. et al. (2022)	Inter-rater reliability	VMI: ICC 0.941	+
[36]	Fulceri, F. et al. (2019)	Not reported	Not reported	Not reported
[37]	Alsaedi, RH. et al. (2020)	Not reported	Not reported	Not reported
[38]	Bricout, VA. et al. (2019)	Not reported	Not reported	Not reported
[39]	Liu, T. et al. (2013)	Not reported	Not reported	Not reported
[40]	Yang, Y. C. et al. (2019)	Not reported	Not reported	Not reported
[41]	Crippa et al. (2021)	Not reported	Not reported	Not reported
[42]	Vanvuchelen (2007)	Not reported	Not reported	Not reported
[43]	Provost (2007)	Inter-rater reliability	PDMS-2: ICC = 0.98	+
[44]	Biffi et al. (2018)	Not reported	Not reported	Not reported
[45]	De Francesco et al. (2023)	Not reported	Not reported	Not reported
[46]	Allen et al. (2017)	Intra-rater reliability	TGMD-3 Traditional: ICC = 0.95 TGDM-3 Visual: ICC = 0.95	+ +
[47]	Staples & Reid (2010)	Not reported	Not reported	Not reported
[48]	Martel et al. (2024)	Not reported	Not reported	Not reported
[49]	Kalfiřt et al. (2023)	Not reported	Not reported	Not reported
[50]	Kochav-Lev et al. (2023)	Not reported	Not reported	Not reported
[51]	Martín-Díaz et al. (2024)	Not reported	Not reported	Not reported

**Table 3:** Standardized tests used in the included articles to assess motor skills in ASD individuals.

Assessment Name	Variable Assessed	Age Range	Assessment Context	Citation
Movement Assessment Battery for Children—Second Edition (MABC-2)	Manual dexterity, aiming and catching, balance	3–16	Structured probes	Bricout et al. (2019) [38] Green et al. (2009) [30]; Odeh et al. (2022) [32]; Miller et al. (2021) [33]; Ament et al. (2015) [34]; Faber et al. (2022) [35]; Liu et al. (2013) [39]; Crippa et al. (2021) [41]; Vanvuchelen et al. (2007) [42]; Biffi et al. (2018) [44]; De Francesco et al. (2023) [45]; Martel et al. (2024) [48]
Bruininks–Oseretsky Test of Motor Proficiency, Second Edition (BOT-2)	Fine motor precision, fine motor integration, manual dexterity, bilateral, coordination, balance, running speed and agility, upper-limb coordination, strength	4–21	Structured probes	Kaur et al. (2018) [31]; Odeh et al. (2022) [32]; Alsaedi et al. (2020) [37]; Kalfirt et al. (2023) [49]; Martín-Díaz et al. (2024) [51]
Peabody Developmental Motor Scales-Second Edition (PDMS-2)	Reflexes, stationary, locomotion, object manipulation, grasping, visual–motor integration.	Birth-5	Structured probes	Fulceri et al. (2019) [36]; Yang et al. (2019) [40]; Vanvuchelen et al. (2007) [42]; Provost et al. (2007) [43]
Test of Gross Motor Development (TGMD)	Locomotor skills: running, jumping, walking on a line, crawling, hurdling. Object control skills: throwing, catching, kicking, bouncing, dribbling	3–10	Structured probes	Allen et al. (2017) [46]; Staples & Reid (2010) [47]
Alberta Infant Motor Scale (AIMS)	Gross motor development, postural control, movement quality	0–18 months	Standardized observational assessment	Kochav-Lev et al. (2023) [50]

**Table 4:** Characteristics of the studies.

References		Study Design	Sample Size	M:F	Years Range (M; SD)	Outcome Measures	Aims	Results
[30]	Green, D. et al. (2009)	observational study	47 ASD 43 broader autistic spectrum disorder	7.4:1	10–14 (M 11.33 ± 0.83)	MABC-2; DCDQ	Explore the degree of impairment in movement skills in autistic children and a wide IQ range.	Among autistic children, 79% showed marked motor deficits on the M-ABC test, and an additional 10% had borderline difficulties. Children diagnosed with infantile autism showed more motor difficulties than those with a broader autistic profile, and children with an IQ below 70 were more affected than those with an IQ above 70.
[31]	Kaur, M. et al. (2018)	qualitative observational study	12 HASD 12 LASD 12 TD	3:9 0:12 2:10	5–12 (M 7.44 ± 0.57) 5–12 (M 8.74 ± 0.59) 5–12 (M 7.75 ± 0.55)	BOT-2; SIPT	Study measures for the comprehensive profiling of the motor system in autistic children.	The BOT-2 revealed a significant group effect for the composite body coordination, composite fine manual, and manual dexterity scores. Both ASD groups scored lower than the TD group on all BOT-2 outcome measures. Compared with the differences between the two ASD groups, the LASD group scored lower than the HASD group on the BOT-SF and body coordination subtests. Both groups demonstrated similarly poor performance on the composite fine manual and manual dexterity subtests.
[32]	Odeh, C.E., et al. (2022)	observational study	12 ASD 12 TD	11:1 11:1	5–12 (M 8.71 ± 1.69) 5–11 (8.74 ± 2.42)	BOT-2, MABC-2	The purpose of this preliminary study was to establish a robust motor profile in autistic children across a wider range of motor skills.	In this study, autistic children demonstrated deficits in the performance of tasks targeting strength, speed, agility, coordination, and both static and dynamic balance. Furthermore, differences were also found between the MABC-2 and BOT-2 scores for the same subtest and aiming and catching tasks.
[33]	Miller, HL. et al. (2021)	retrospective study	43 ASD 18 DCD	37:6 7:2	5–19 (M 11.05 ± 3.6) 8–14 (M 11.05 ± 3.6)	DCDQ; MABC-2; Beery VMI	The study aimed to determine whether motor problems in ASD represent the possible co-occurrence of DCD.	Over 97% of cases in our ASD group met DSM-5 Criterion A based on MABC-2 scores; over 92% met Criterion B based on DCD-Q scores.
[34]	Ament, K. et al. (2015)	observational study	56 ASD 63 ADHD 81 TD	8:1 6:1 23:4	(M 10.27 ± 1,8) (M 9.98 ± 1.34) (M 10.31 ± 1.18)	MABC-2	The study aims to compare motor functioning among three groups: autistic children, ADHD children, and TD children, in order to better define motor deficits in these clinical groups and understand whether motor deficits assist in distinguishing between clinical groups.	Comparing the typically developing group and the developmental disability (DD) group revealed that all three MABC subscale scores were significantly negatively associated with having a DD. For manual dexterity, the mean is 4.88; aiming/catching is 6.52; and balance is 4.82. Impairments in motor skills that require the coupling of visual and temporal feedback to regulate movement appear deficient in ASD.
[35]	Faber, L. et al. (2022)	observational study	17 ASD 17 TD	1:0.24	9.83–15.13 ASD; 10.51–14.80 TD	MABC-2, VMI, VP	The first aim of this study was to examine motor skills, visual perception, and visual–motor integration of autistic children and youth in comparison with age- and gender-matched individuals without ASD. The second aim was to determine if there was an association between motor skills, visual perception, and VMI among children and youth with and without ASD.	Autistic children and youth showed lower overall motor skills, particularly in aiming and catching and balance subscales of the MABC-2, compared with neurotypical peers. No significant associations were found between motor skills, visual perception, and visual–motor integration in the ASD group, suggesting these are not underlying mechanisms of motor deficits.
[36]	Fulceri, F. et al. (2019)	qualitative observational study	32 ASD		2.5–5 (M 48.5 ± 8.8)	PDMS-2;	This study aimed to explore the associations between motor skills and clinical/developmental features in a sample of ASD preschoolers through ANNs (artificial neural networks).	The findings revealed that poor motor skills were a common clinical feature of ASD preschoolers, relating both to the high level of repetitive behaviors and to the low level of expressive language. Total QM, gross QM, and fine QM mean scores were placed into the “Poor class”; any child that had an individual total QM score was in the Average class; one child and two children had, respectively, gross QM and fine QM in the Average class. Almost all subscale mean scores were in the “Below Average class” or the “Poor class.”
[37]	Alsaedi, RH. et al. (2020)	observational study	119 ASD 30 TD	24:6 4:1	6–12 (M 8.7 ± NS)	BOT-2	This study aims to determine the prevalence, severity, and nature of the motor abnormalities seen in ASD children as well as to elucidate the associated developmental profiles.	The results revealed the high prevalence of motor abnormalities in the ASD group compared with normative data derived from the BOT-2 manual. Furthermore, the findings suggest that the age variable may influence the overall motor performance of ASD children and that children’s motor abnormalities may decrease with maturation.
[38]	Bricout, VA. et al. (2019)	observational study	22 ASD 20 TD	22:0 20:0	8–12 (M 10.7 ± 1.3) 8–12 (M 10.0 ± 1.6)	EUROFIT; PANESS; M-ABC	The present study provides a physical fitness profile of autistic children. The second aim of this study was to identify which motor tests best discriminate between autistic and non-autistic children.	In the ASD group, flexibility, explosive power, and strength scores were significantly lower compared with the control group. The results also showed significant difficulties in ASD children regarding dexterity and ball skills. The principal component analysis and agglomerative hierarchical cluster analysis allowed for the classification of children based on motor tests, correctly distinguishing clusters between children with and without motor impairments. Autistic children showed significantly lower MABC scores compared with the control group. Major deficits were observed in manual dexterity and ball skills, while balance differences were not statistically significant.
[39]	Liu, T. et al. (2013)	retrospective study	30 ASD 30 TD	13:2 8:7	3–16 (M 7.96 ± 3.14) 3–16 (M 7.44 ± 2.36)	MABC-2	The aim of this study was to investigate the fine and gross motor performance of autistic children and typically developing children of the same age.	According to the results obtained, autistic children presented delays in the performance of both fine and gross motor skills on the MABC-2 compared with typically developing children of the same age. A total of 77% of autistic children were in the red zone, 3% were in amber zone, and 20% were in the green zone.
[40]	Yang, Y. C. et al. (2019)	prospective observational study	15 ASD 15 TD 15 VLBW-PT	13:2 3:2 3:2	2.5–3 (M 2.5 ± NS)	PDMS-2;	This study aims to compare cognitive, motor, and behavioral developments and free-play movement performance in ASD toddlers who were full term (FT-ASD), toddlers who were full term and are typically developing (FT-TD), and toddlers who were born preterm and had a very low birth weight (VLBW-PT).	Children with FT-ASD showed lower cognitive and motor scores and a higher degree of behavioral problems than children with FT-TD or VLBW-PT. Furthermore, in free play, children with FTASD performed a higher degree of rotation speed, a higher movement time, and a higher frequency of movement towards the peripheral region than children with FT-TD or VLBW-PT.
[41]	Crippa et al. (2021)	observational study	98 ASD 98 TD	3,3:1	3–11 (M 7.21 ± 2.41)	MABC2-DCDQ	To investigate sex-related differences in the motor profiles of autistic boys and girls aged 3 to 11 years using a multi-method approach.	Autistic children showed significantly lower scores than typically developing peers on all subscales of the MABC-2: manual dexterity, aiming and grasping, and balance. No interaction between diagnosis and sex was found, suggesting similar motor profiles in autistic boys and girls. A sex-related difference was observed in one kinematic characteristic: females showed reduced motor anticipation compared with males.
[42]	Vanvuchelen (2007)	observational study	8 low-functioning with autism (LFA) 13 with mental retardation (MR) 17 high-functioning with autism (HFA) 17 typically developing (TD)		5.1–10.6 (M 8.04 ± 0.84)	MABC-PDMS	The aim was to investigate whether the difficulties in gestural imitation of children with autism depended on a cognitive–representational or perceptual–motor deficit through the manipulation of task variables in two groups of autistic subjects and one group with typical development.	The results revealed that all autistic children, with and without mental disorders, had more problems imitating non-significant gestures than significant gestures compared with non-autistic controls. LFA performed significantly worse than RM on the motor test and imitation tasks. The HFA performed significantly worse than the TD in the motor test, but not in the imitation tasks, with the exception of non-significant gestures. This study supports the idea that underlying the difficulties in gestural imitation of children with autism is a perceptual–motor impairment.
[43]	Provost (2007)	observational study	19 ASD 19 DD	30:8	1–3 (M 2.53 ± 0.38)	PDMS	The aim of the study was to compare the levels of gross motor (GM) and fine motor (FM) development in toddler-aged autistic children with the GM and FM development levels of children with developmental delay (DD) who are not autistic.	The results showed that autistic children had generally similar levels of GM and FM development. The motor profiles of autistic children did not differ significantly from those of children with DD.
[44]	Biffi et al. (2018)	comparative experimental study	15 ASD 16 TD	14:1 15:1	7.4–11.6 (M 9.91 ± 1.44)	MABC2-DCDQ	The objective is to describe the gait pattern and motor performance of school-age autistic children, not treated with medication, compared with a sample of the same gender and age with TD.	The results indicated an abnormal gait pattern, in which autistic children tend to increase their locomotion stability. No difference between the groups regarding the spatio-temporal parameters. A significant difference was observed in walking speed and a slightly reduced stride length and an increase in the stance phase of the gait cycle. Motor skills measured by tests showed that the ASD group had lower scores on MABC2 manual pointing and grasping, balance and total score, and lower DCDQ scores than TD.
[45]	De Francesco et al. (2023)	observational study	25 ASD 25 ADHD 25 TD	21:4 21:4 21:4	7–13 (10.23 ± 1.22)	DCDQ; MABC2; NEPSY-II	The aim was to discriminate through multimodal assessment the motor skills of autistic children, ADHD children, and peers with typical development.	The results showed that the DCDQ questionnaire and motor imitation skills were the best predictors of autism when compared with TD children, with an accuracy of 87.2%. Differences between ADHD and TD children in the DCDQ and motor skills differentiated the two groups of participants with an accuracy level of 73.5%. The difference between the groups was observed in the MABC2 subscales of pointing and catching, which predicted the status of the autistic group with an accuracy of 70%.
[46]	Allen et al. (2017)	observational study	14 ASD 21 TD	12:9 5:2	4–10 (M 7.13 ± 1.96)	TGMD-3	Evaluate the reliability and validity of the TGDM-3 in two conditions: with the traditional protocol and with the visual support protocol.	Results confirm the traditional TGMD-3 protocol and the TGMD-3 visual support protocol as reliable measures in measuring gross motor performance children with typical development and autistic children. The test has internal consistency and excellent levels of test–retest, inter-rater, and intra-rater reliability using both tests and both experimental groups.
[47]	Staples & Reid (2010)	observational study	25 ASD 25 chronological age-matched 22 developmentally matched group 19 mental age-matched group	21:4 21:4 18:4 16:3	9–18 (M 11.15 NS)	TGMD-2	The study compares the motor skill performance of autistic children with three types of typically developing groups individually matched on specific developmental variables: chronological age, motor ability, and cognitive development.	Motor skill performance in ASD is significantly delayed in late childhood.
[48]	Martel et al. (2024)	observational study	14 ASD 14 DCD 14 TD	6:1 4:3 2.5:1	7–12 (M 10.4 ± 0.29)	MABC-2	To compare motor deficits in autistic children with those in children with DCD and TD in order to identify distinctive features between the three groups.	ASD children demonstrate specific motor deficits, distinct from those of DCD. TD children showed significantly better scores on all motor measures compared with ASD and DCD groups.
[49]	Kalfirt et al. (2023)	observational study	17 ASD 15 TD	32:0	9–13 (ASD: M 11.1 ± 1.0; TD: M 11.0 ± 0.5)	BOT-2; Heart Rate Variability; Arterial Stiffness	To compare motor skills, heart rate variability, and arterial stiffness between autistic children and TD.	ASD children showed significant deficits in manual coordination, strength, agility, and total motor composite compared with TD. HRV: Significantly reduced in ASD, indicating autonomic dysfunction. Arterial stiffness: No significant differences between groups.
[50]	Kochav-Lev et al. (2023)	observational study	15,185 MDD; 388 ASD and MDD	3.3:1	0–2	AIMS	To evaluate the use of AIMS for early detection of motor developmental delay (MDD) as an indicator for ASD in infants.	Odds ratio for ASD with MDD: 4.1 (95% CI: 3.6–4.6). A total of 87% of ASD infants showed MDD or suspected MDD on AIMS. A total of 98% of motor delay referrals confirmed as MDD or suspected MDD. Early referral (0–6 months): there was 39% MDD detection, increasing to 68% at older ages.
[51]	Martín-Díaz et al. (2024)	cross-sectional observational study	50 ASD 50 TD	6:1 6:1	(ASD M 9.54 ± 3.09) (TD M 9.54 ± 3.09)	SFBOT-2; TUG	To compare static and dynamic balance, postural control, and motor skills between autistic children and adolescents and those with TD, using specific assessment tests. To examine the potential correlation between the scores of the assessment tools used in the study among children and adolescents with ASD.	The ASD group showed significantly worse performance in TUG, SFBOT-2, and PBS. Motor deficits were most evident in strength, manual dexterity, agility, and balance. Weak correlations were found between TUG and SFBOT-2/PBS; moderate correlation between SFBOT-2 and PBS. Monopodal support with hands on hips showed the largest difference in PBS.

Legend: AIMS—Alberta Infant Motor Scale; ASD—autism spectrum disorder; Beery VMI—Beery-Buktenica Developmental Test of Visual-Motor Integration; BOT-2—Bruininks–Oseretsky Test of Motor Proficiency, Second Edition; BOT-2 Brief Form BOT-2 BF; DCD—developmental coordination disorder; DCDQ—Developmental Coordination Disorder Questionnaire; DD—developmental disability; Exam; HRV—heart rate variability; IQ—intelligence quotient; LFA—low-functioning autism; MABC-2—Movement Assessment Battery for Children, Second Edition; MDD—motor developmental delay; PANESS—Physical and Neurological Examination for Subtle Signs; PDMS-2—Peabody Developmental Motor Scales, Second Edition; TGMD-2/TGMD-3—Test of Gross Motor Development, Second and Third Edition; TUG—Timed Up and Go Test; VMI—Developmental Test of Visual-Motor Integration; VMI-6—Beery-Buktenica Developmental Test of Visual-Motor Integration, Sixth Edition; Vineland-3—Vineland Adaptive Behavior Scales, Third Edition; VP—visual perception; WOS—Walk or Sit Test; Ignite Challenge Scale—Scale to Assess Motor Control, Coordination, and Motor Planning in ASD; pediatric balance scale (PBS); neurotypical development (TD).

**Table 5:** QUADAS-2 risk of bias and applicability summary. Summary of risk of bias and applicability concerns across the 22 included studies, assessed using the QUADAS-2 tool.

**Study**	**Risk of Bias**				**Applicability Concerns**		
	**Patient Selection**	**Index Test**	**Reference Standard**	**Flow and Timing**	**Patient Selection**	**Index Test**	**Reference Standard**
[48] Martel, 2024	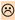	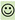	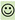	?	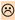	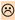	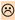
[51] Martín-Díaz, 2024	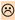	?	?	?	?	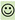	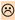
[45] De Francesco, 2023	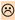	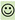	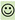	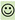	?	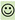	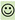
[49] Kalfirt, 2023	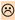	?	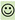	?	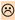	?	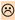
[50] Kochav-Lev, 2023	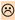	?	?	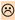	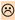	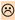	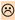
[35] Faber, 2022	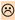	?	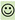	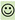	?	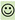	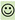
[32] Odeh, 2022	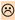	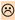	?	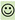	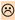	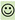	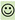
[41] Crippa, 2021	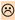	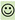	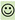	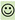	?	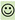	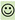
[33] Miller, 2021	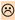	?	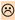	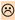	?	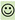	?
[37] Alsaedi, 2020	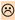	?	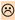	?	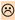	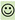	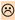
[38] Bricout, 2019	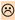	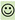	?	?	?	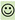	?
[36] Fulceri, 2019	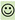	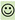	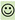	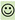	?	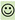	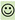
[40] Yang, 2019	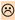	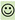	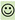	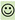	?	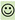	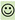
[44] Biffi, 2018	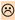	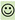	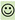	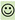	?	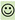	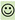
[31] Kaur, 2018	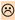	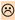	?	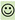	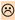	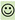	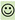
[46] Allen, 2017	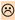	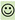	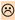	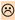	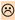	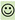	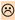
[34] Ament, 2015	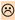	?	?	?	?	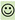	?
[39] Liu, 2013	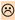	?	?	?	?	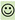	?
[30] Green, 2009	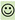	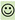	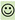	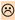	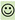	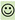	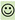
[47] Staples, 2009	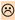	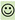	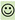	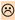	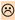	?	?
[43] Provost, 2007	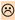	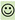	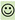	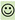	?	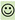	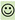
[42] Vanvuchelen, 2007	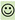	?	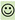	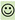	?	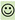	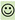

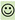
 Low risk; 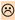 high risk; ? unclear risk.

## Data Availability

No new data were created or analyzed in this study. Data sharing is not applicable to this article.

## Supplementary Materials

The following supporting information can be downloaded at: https://www.mdpi.com/article/10.3390/diagnostics15172118/s1, Supplementary Table S1. PRISMA 2020 Checklist.
